# Social determinants associated with Zika virus infection in pregnant women

**DOI:** 10.1371/journal.pntd.0009612

**Published:** 2021-07-30

**Authors:** Nivison Nery, Juan P. Aguilar Ticona, Claudia Gambrah, Simon Doss-Gollin, Adeolu Aromolaran, Valmir Rastely-Júnior, Millani Lessa, Gielson A. Sacramento, Jaqueline S. Cruz, Daiana de Oliveira, Laiara Lopes dos Santos, Crislaine G. da Silva, Viviane F. Botosso, Camila P. Soares, Danielle Bastos Araujo, Danielle B. Oliveira, Rubens Prince dos Santos Alves, Robert Andreata-Santos, Edison L. Durigon, Luís Carlos de Souza Ferreira, Elsio A. Wunder, Ricardo Khouri, Jamary Oliveira-Filho, Isadora C. de Siqueira, Antônio R. P. Almeida, Mitermayer G. Reis, Albert I. Ko, Federico Costa

**Affiliations:** 1 Instituto de Saúde Coletiva, Universidade Federal da Bahia, Salvador, Brazil; 2 Instituto Gonçalo Moniz, Fundação Oswaldo Cruz, Ministério da Saúde, Salvador, Brazil; 3 Department of Epidemiology of Microbial Diseases, Yale School of Public Health, New Haven, Connecticut, United States of America; 4 Precision Vaccines Program, Division of Infectious Diseases, Boston Children’s Hospital, Boston, Massachusetts, United States of America; 5 Faculdade de Medicina da Bahia, Universidade Federal da Bahia, Salvador, BA–Brazil; 6 Development and Innovation Center, Laboratory of Virology, Butantan Institute, São Paulo, Brazil; 7 Departamento de Microbiologia. Instituto de Ciências Biomédicas, Universidade de São Paulo, São Paulo, Brazil; 8 Laboratório de Desenvolvimento de Vacinas, Departamento de Microbiologia, Instituto de Ciências Biomédicas, Universidade de São Paulo, São Paulo, Brazil; 9 Scientific Platform Pasteur USP, São Paulo, Brazil; Fundaçao Oswaldo Cruz, BRAZIL

## Abstract

This study aims to describe the sociodemographic determinants associated with exposure to Zika Virus (ZIKV) in pregnant women during the 2015–2016 epidemic in Salvador, Brazil.

**Methods:**

We recruited women who gave birth between October 2015 and January 2016 to a cross-sectional study at a referral maternity hospital in Salvador, Brazil. We collected information on their demographic, socioeconomic, and clinical characteristics, and evaluated their ZIKV exposure using a plaque reduction neutralization test. Logistic regression was then used to assess the relationship between these social determinants and ZIKV exposure status.

**Results:**

We included 469 pregnant women, of whom 61% had a positive ZIKV result. Multivariate analysis found that lower education (adjusted Prevalence Rate [aPR] 1.21; 95%CI 1.04–1.35) and food insecurity (aPR 1.17; 95%CI 1.01–1.30) were positively associated with ZIKV exposure. Additionally, age was negatively associated with the infection risk (aPR 0.99; 95%CI 0.97–0.998).

**Conclusion:**

Eve after controlling for age, differences in key social determinants, as education and food security, were associated with the risk of ZIKV infection among pregnant women in Brazil. Our findings elucidate risk factors that can be targeted by future interventions to reduce the impact of ZIKV infection in this vulnerable population.

## Author summary

The Zika virus (ZIKV) epidemic in Brazil has intensified global concern about congenital defects associated with intrauterine exposure. Social determinants are factors that reinforce and contribute to the transmission and spread of ZIKV as well as other arboviruses like Dengue. We performed a cross-sectional study to describe the prevalence of ZIKV and the contribution of social determinants to transmission among pregnant women during the 2015–2016 ZIKV epidemic in Salvador, Brazil. We found that 61% of pregnant women were ZIKV seropositive. We also found that lower education level, food insecurity and lower maternal age were associated with higher ZIKV infection risk. These findings contribute to understanding the role of social determinants in ZIKV transmission, providing key social factors that can be combined with pre-existing tactics (vector control and environmental improvement) to create policies and interventions which reduce social inequalities and risk of infection in vulnerable populations like pregnant women.

## Introduction

Zika virus (ZIKV) has become an emerging global public health problem [1–3] with 87 countries reporting ZIKV outbreaks in 2019 [4]. The ZIKV epidemic which occurred in Brazil between 2015 and 2016 is currently the largest of these on record. The magnitude of ZIKV presence in Brazil may be related to the extensive habitat and abundance of the *Aedes aegypti* mosquito, which is its main vector [5,6], as well as to the vulnerability of the local population due to their lack of prior immunity [7]. Around 80% of people infected with ZIKV are asymptomatic while the rest present symptoms such as headache, fever, arthralgia and rash that can last between a few days and a week [8]. The paradigm shifts, however, in the case of vertical transmission, and studies during the Brazilian epidemic brought to light significant maternal-child health issues associated with ZIKV infection. [9,10]. Intrauterine exposure to ZIKV can result in Congenital Zika Syndrome (CZS), which includes a range of physical and neurological impairments, such as microcephaly [9–11].

The spread of the ZIKV epidemic across Brazil was not uniform. Although the disease was registered in most Brazilian states, the epicenter of this disease was in the northeastern region, where the highest rates of congenital abnormalities were registered [12,13]. In Brazil between 2015 to 2017 were registered 2,751 cases of CZS among them 69.5% were reported in the northeast region [14]. One possible explanation for this disease prevalence heterogeneity within the country may be the role of social and environmental factors which vary across regions. [15,16]. Social inequalities such as food insecurity, unemployment and low schooling have been linked to problems with infrastructure and sanitation which may allow the spread and proliferation of these diseases [17]. This has been previously shown in the case of dengue virus (DENV) infection, another flavivirus, closely related to ZIKV, for which prior studies suggest that these variables help to explain the impact and distribution of the disease [18]. In fact, these social determinants are known to correlate with increased susceptibility to numerous diseases, many of which have been classified as neglected, meaning that they primarily affect people living in poverty around the world [19]. As a result, it is not surprising that in Brazil significant disparities have been observed in the demographic characteristics of mothers of children born with CZS, 80% of whom are young, identify as black or brown, have a low income, and live in less well-developed regions of the country [20].

It has been projected that more than half of the world’s population will live in tropical regions by 2030 [21]. Therefore it is urgently necessary to elucidate the social and environmental determinants associated with ZIKV infection, in order to improve future strategies to control ZIKV and other emerging arboviruses in these tropical regions. In particular, we should focus on identifying these risk factors in vulnerable populations, especially pregnant women, in order to construct improved prenatal care recommendations for them in the face of future emerging diseases. Furthermore, understanding the social determinants of ZIKV infection will also facilitate the targeting of other effective disease-prevention measures, like vector control, to these vulnerable and high-risk groups. In this study, we assess the sociodemographic, economic, and social factors associated with ZIKV infection, describe the prevalence and the most common clinical characteristics of this infection in women who gave birth at a referral hospital in the city of Salvador, Brazil.

## Methods

### Ethics statement

The project was approved by the Human Research Ethics Committee of The General Hospital Roberto Santos, through the Certificate of Presentation for Ethical Appreciation (CAAE) number 5344216.1.1001.5028 and Yale University (1603017343). All women who were eligible and participated in the study, read and signed the Free and Informed Consent Form (ICF) before the collection of clinical data and biological samples.

### Study site and Socioeconomic Survey

We performed a cross-sectional study at Roberto Santos General Hospital (*Hospital Geral Roberto Santos*—HGRS), a referral hospital in Salvador, Brazil. Salvador (population, 2.9 million in 2020) is located in the state of Bahia in northeastern Brazil [22]. HGRS is the largest tertiary referral health center in the state, with 640 beds and an average of 1,300 hospitalizations per month. HGRS has an obstetric center that is responsible for 140 births per month, and which receives patients with high-risk pregnancies from throughout the nearby region.

In this study we recruited pregnant women who resided in Salvador, Brazil and who gave birth at the HGRS obstetric center, between October 1, 2015 and January 31, 2016. Women were enrolled during the postpartum period or during the second year of their child’s life. A structured questionnaire was used by a trained team (including physicians and nurses) to survey participants about their demographic, socioeconomic and clinical history (including symptoms related to ZIKV during pregnancy). We measured food insecurity during pregnancy using a two-item screening method developed by Hager E.R et al [23]. Participants were asked whether the following statements were true or false: 1) Within the pregnancy we worried whether our food would run out before we got money to buy more, and 2) Within the pregnancy the food we bought just did not last and we did not have money to get more. Hager et al. reported that an affirmative response to at least one of these two items showed 97% sensitivity and 83% specificity and was therefore highly effective at identifying food insecurity. We categorized participants as “at risk of food insecurity” if they answered “yes” to either of the screening questions. Additionally, participants categorized as at risk of food insecurity by the screening, completed an short 12-item questionnaire from the Brazilian Food Insecurity Scale (Escala Brasileira de Insegurança Alimentar – EBIA)[24]. All information about the pregnancy period was collected retrospectively during postpartum or their child’s second year of life. The subject’s clinical history and prenatal Brazilian monitoring program card were used to reduce memory bias.

### ZIKV exposure

ZIKV exposure was determined by Plaque Reduction Neutralization Test (PRNT) [19]. PRNT was performed on serum samples collected at birth and during mother-child follow ups between 2 to 3 years after birth. The antibody titer for PRNT was defined based on a 50% reduction in inhibition of virus inoculum (PRNT_50_). Serum samples were considered positive when the antibody titers were ≥ 20 for ZIKV. PRNT_50_ assays were also performed for the four dengue serotypes (DENV 1, DENV 2, DENV 3 and DENV 4) in a subgroup of samples collected during the delivery period, in order to assess the possibility of cross reaction between the two flaviviruses. For mothers from whom blood samples were collected more than once, PRNT results were compared in order to validate the results of the assay.

### Data analysis

Data collection was performed using electronic questionnaires, and responses were stored using the REDCap (Research Electronic Data Capture) data collection and data management system and saved on the Gonçalo Moniz Institute (IGM) server. Statistical analyses were performed using RStudio software (RStudio Version 1.2.5033).

We summarized the data using descriptive statistics. Categorical data were compared using Fisher’s exact test. Quantitative data were compared using the Mann-Whitney test. Agreement analysis between PRNT results at birth and during the follow up (2 to 3 years after the birth) were analyzed using Cohen’s Kappa coefficient of agreement and 95% Confidence Interval (95% CI). Additionally, Spearman’s correlation coefficients were calculated to evaluate the correlation between both PRNT results. Bivariate binomial logistic regressions were performed to assess the risk factors and the symptoms associated with infection, and those with a significance level <0.20 were included in the multivariate models. We used Prevalence Ratio (PR) to measure the associations, with a 95% CI and a p-value <0.05 was considered statistically significant.

## Results

**Fig 1:**
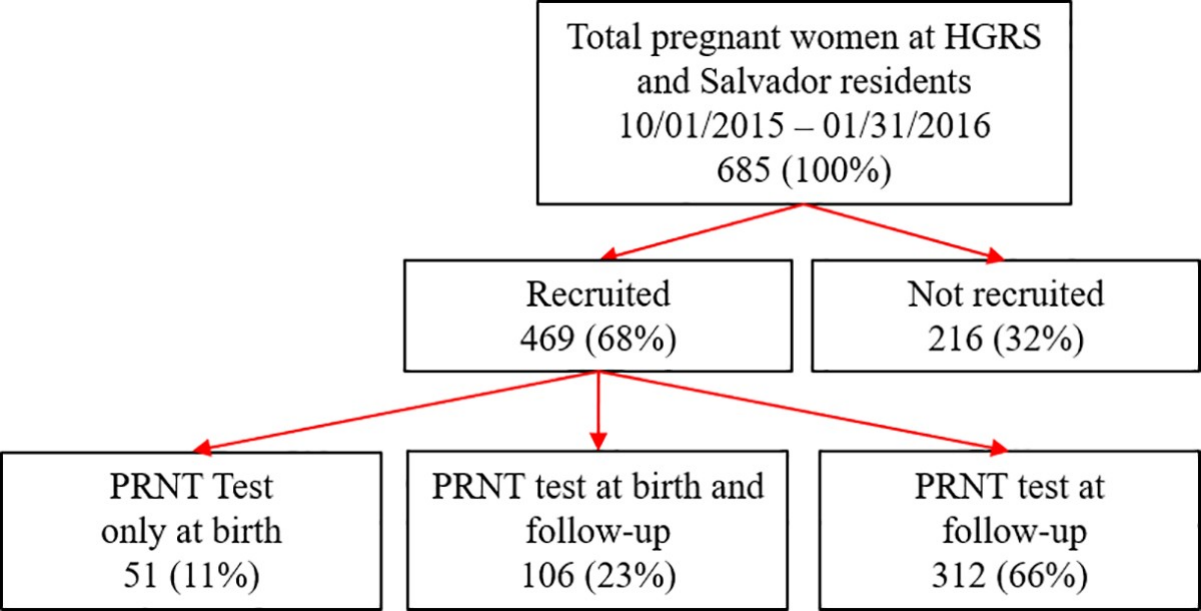
Study flowchart.

During the period of the study, from October 1^st^ 2015 to January 31^st^ 2016, HGRS provided delivery service to 685 pregnant women from Salvador, Brazil. We enrolled 469 (68,5%) of these women in our study, of whom 157 (33.5%) were enrolled when they gave birth and 312 (66.5%) were enrolled two years later at the time of their child’s developmental follow up appointment (Fig 1).

**Fig 2:**
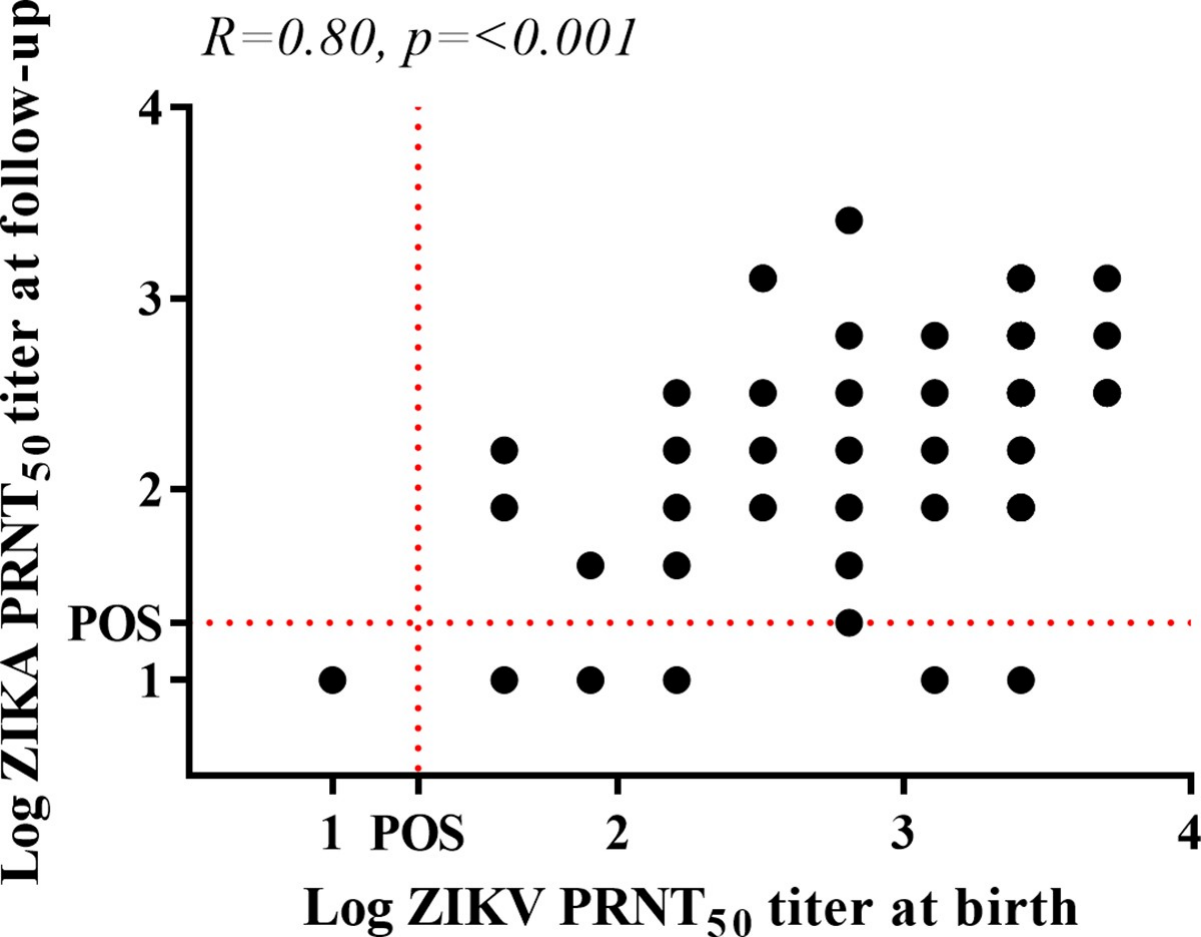
Correlation between ZIKV PRNT_50_ titer at birth and ZIKV PRNT_50_ titer at follow up between 106 women with samples at birth and samples at follow-up.

Among the 157 women with a ZIKV PRNT result at delivery, 106 (22.6%) women also had a ZIKV PRNT result during the follow up (2 to 3 years after delivery). This subsample was used to evaluate the seroconcordance and confirm the correlation between the ZIKV PRNT results at delivery and at follow up. We found a 93% concordance (74 positives and 25 negatives) between both different sample collection time points (Kappa = 0.83; 95% CI 0.77–0.98), as well as a significant positive correlation β = 0.75 (p <0.01) between quantitative PRNT values in the two periods (Fig 2). The overall evaluation revealed ZIKV neutralizing antibodies (nAb) in 287 samples, indicating a seroprevalence of 61.2%, as shown in Table 1 alongside the demographic, socioeconomic, and clinical characteristics of the interviewed women.

**Table 1:** Demographic and clinical characteristics of pregnant women in the study.

Characteristic	No. responses	Total (N = 469)^a^	Zika positive (N = 287)^a^	Zika negative (N = 182)^a^	p-value^b^
**Demographics**					
Age (years)	468	26 (20–32)	25 (20–31)	27 (21–33)	**0.017**
Ethnicity	414				0.95
White		16 (3.9)	10 (3.9)	6 (3.9)	
Black		212 (51)	134 (52)	78 (50)	
Brown		182 (44)	113 (44)	69 (45)	
Others		4 (1.0)	2 (0.8)	2 (1.3)	
Education	417				**0.016**
Less than 9 years (No high school)		292 (70)	169 (66)	123 (77)	
More than 9 years (Some high school)		125 (30)	88 (34)	37 (23)	
Number of residents in the house	398	3 (2–4)	3 (2–4)	3 (2–4)	0.59
Worked before pregnancy	414	194 (47)	119 (47)	75 (47)	>0.99
Worked with a formal contract	325	96 (30)	53 (27)	43 (33)	0.33
Dissatisfied with family income	409	233 (57)	146 (59)	87 (54)	0.41
Income	263				>0.99
below minimum wage		27 (10)	16 (10)	11 (10)	
above minimum wage		236 (90)	142 (90)	94 (90)	
Receives government financial assistance	102	61 (60)	44 (63)	17 (53)	0.39
**Food insecurity screening**					
At risk of food insecurity	409	247 (60)	158 (63)	89 (56)	0.12
**Diagnoses and exposures**					
Poor self-perceived health	412	21 (5.1)	16 (6.3)	5 (3.1)	0.17
Pre-existing diseases	417	92 (22)	66 (25)	26 (16)	**0.038**
Sexually transmitted disease	411	27 (6.6)	18 (7.0)	9 (5.8)	0.69
TORCHS^c^	469	10 (2.1)	6 (2.1)	4 (2.2)	>0.99
Symptoms during pregnancy					
Rash	469	51 (11)	49 (17)	2 (1.1)	**<0.001**
Fever	469	45 (9.6)	41 (14)	4 (2.2)	**<0.001**
Cough	469	15 (3.2)	12 (4.2)	3 (1.6)	0.18
Headache	469	48 (10)	44 (15)	4 (2.2)	**<0.001**
Arthralgia	469	49 (10)	46 (16)	3 (1.6)	**<0.001**
Muscle ache	469	46 (9.8)	43 (15)	3 (1.6)	**<0.001**
Conjunctivitis	469	9 (1.9)	9 (3.1)	0 (0)	**0.014**
Alcohol use during the pregnancy	389	103 (26)	71 (29)	32 (22)	0.12
Smoking during the pregnancy	389	24 (6.2)	17 (7.0)	7 (4.8)	0.51

^a^Median (IQR); n (%)

^b^Welch Two Sample t-test; Fisher’s exact test

^c^TORCH, Toxoplasmosis, Rubella, Cytomegalovirus, Herpes infections and Others

Bold numbers indicate statistically significant differences (p <0.05) between ZIKV positive and ZIKV negative women

Most of the pregnant women were young adults with a median age of 25 years (IQR 20–31), 212 (51.2%) self-identified as black, and 125 (30.0%) had less than 9 years of schooling (ie. had not attended high school). We found that pregnant women with a positive ZIKV PRNT result were more likely to be younger [ZIKV (+) 25 years old vs ZIKV (-) 27 years old; p = 0.017] and less educated [ZIKV (+) 34.2% vs ZIKV (-) 22.5%; p = 0.016]. Among 247 (52.6%) pregnant women meeting the standard for food insecurity based on our screening, 158 (54.9%) had a positive ZIKV PRNT result and 90 (47.1%) had a negative ZIKV result, with a p-value of 0.127 (Table 1). Additionally, within the subsample of participants who completed the full food insecurity, a higher proportion of answers associated with food insecurity was shown in participants with a positive ZIKV PRNT, however this difference was not statistically significant (S1 Table). Our multivariate analysis showed that age in years (aPR 0.99; CI 0.97–0.998), schooling (aPR 1.21; CI 1.04–1 .35) and food insecurity (aPR 1.17; CI 1.01–1 .30) were all associated with increased risk of a positive ZIKV PRNT result (Table 2).

**Table 2:** Multivariable analysis of sociodemographic factors associated with a positive ZIKV PRNT_50_ result between 377 women in Brazil.

Characteristics	PR	95% CI	p-value	aPR	95% CI	p-value
Age (years)	0.99	0.98–1^a^	0.017	0.99	0.97–1^b^	0.023
Education	1.19	1.04–1.32	0.016	1.21	1.04–1.35	0.020
Food insecurity risk at screening	1.13	0.96–1.34	0.120	1.17	1.01–1.30	0.038

Prevalence Ratio (PR), Adjusted prevalence ratio (aPR)

^a^ Upper limit of CI = 0.997

^b^ Upper limit of CI = 0.998

We also evaluated the clinical symptoms associated with ZIKV exposure. Unsurprisingly, we found that pregnant women who tested positive for ZIKV reported a higher frequency of symptoms during pregnancy, such as rash [ZIKV (+) 17.1% vs ZIKV (-) 1.1%; p <0.01], fever [ZIKV (+) 14.3% vs ZIKV (-) 2.2%; p <0.01), headache [ZIKV (+) 15.3% vs ZIKV (-) 2.2%; p <0.01), arthralgia [ZIKV (+) 16.0% vs ZIKV (-) 1.6%; p <0.01), muscle pain [ZIKV (+) 15.0% vs ZIKV (-) 1.6%; p <0.01) and conjunctivitis [ZIKV (+) 3.1% vs 0%; p = 0.01) in relation to mothers with negative results. In the multivariate analysis, only rash (aPR 1.52; 1.26–1.62) showed an association with ZIKV infection after adjusting for the presence of arthralgia symptoms (Table 3).

**Table 3:** Multivariable analysis of symptoms during pregnancy in 469 women associated with a positive ZIKV PRNT_50_ result.

Characteristics	PR	95% CI	p-value	aPR	95% CI	p-value
Rash	1.68	1.53–1.87	<0.001	1.52	1.26–1.62	0.008
Arthralgia	1.64	1.47–1.83	<0.001	3.16	0.95–1.36	0.101

Prevalence Ratio (PR), Adjusted prevalence ratio (aPR)

Confidence interval (CI)

## Discussion

During the 2015–2016 Brazilian ZIKV epidemic, the northeast region of the country bore the brunt of the disease burden, however for the role of social and environmental factors in geographical disparities in infection rates remains to be identified [10,25]. This regional phenomenon could also be observed in our study, in a referral obstetric center at HGRS, in Salvador, Northeastern of Brazil. We found that among 469 pregnant women recruited to our study, 61% had been infected with ZIKV. We also found that low education and food insecurity were positively associated with ZIKV exposure, while age in years was negatively associated with ZIKV antibodies.

The large proportion (61%) of pregnant women in our study with PRNT-confirmed ZIKV infections is consistent with the prevalence found in another study in Salvador, which tracked a community cohort in a low income neighborhood, and found a rate of infection of 73% [12] as well as another ZIKV serosurvey which included pregnant women that found a seroprevalence of 63.3% [10]. Emphasizing the contrast between northeastern and southern Brazil, cross sectional studies on other regions of Brazil found significantly lower seroprevalences [26].

We identified significant relationships between maternal ZIKV exposure and two socioeconomic variables: maternal schooling and household food insecurity. Those two determinants of health play a foundational role, not just in determining the risk of ZIKV infection, but in a wide range of public health challenges [27–30]. One possible explanation for the association between low education and higher vulnerability to infection is that education is associated with better knowledge and safety practices regarding infectious diseases [27,28]. This is consistent with several studies analyzing the role of education level in the knowledge, attitudes and practices related to ZIKV and DENV transmission [27–29]. In Belo Horizonte, Brazil, during a seven-year study, seven dengue epidemic waves were recorded, with regions possessing a higher prevalence of adults with low levels of education being the most heavily impacted [27]. Moreover, other studies in two cities of Brazil have found associations between low economic conditions and low educational level was associated with less use preventive practice against Zika and higher ZIKV prevalence [10,31]. But this phenomena is not restricted to regional areas, also it was register in all Brazil territory, where the most affected region was the northeast that it is a lower developed region with lower income rates and lower education levels [32].

Like education level, food insecurity is a social determinant of health which is associated with poverty and low income [33,34]. Moreover, food insecurity and subsequent malnutrition can directly promote human susceptibility to infection diseases, while infectious diseases themselves can sometimes inhibit the body’s digestive processes and exacerbate malnutrition in turn [30]. For example, there is evidence that vitamin A and D supplementation can reduce the risk of DENV infection, while in the context of ZIKV the risk of CZS-associated microcephaly is increased among individuals who fail to consume enough protein in their diets [35,36].

We found that young pregnant women were more likely to have a positive ZIKV PRNT test result. Similar results were found in a study of ZIKV in Puerto Rico, where the incidence rate decreased in older women and those most affected were pregnant women between 20–29 years old [37]. The difference that we found was small, however, with less than a 2-year difference in median age between positive and negative participants. The relatively low contribution of age could be due to the limited age range allowed for by our exlusive enrollment of pregnant women, although previous studies have also failed to identify large differences in ZIKV risk between age groups [10].

We found that ZIKV positive participants reported fever (14%), rash (17%) and other nonspecific symptoms at a higher rate during pregnancy than ZIKV negative participants. This is similar to previous studies which reported that around 80% of ZIKV infected individuals were asymptomatic and that among those who reported symptoms, the principal complaints were rash, fever, arthralgia, myalgia, fatigue, headache, and conjunctivitis [1,38]. Furthermore, in the multivariate analysis, we found that women who reported rash during their pregnancy were 68% more likely to have a positive ZIKV PRNT result. Again, this is consistent with prior literature. For example, among symptomatic cases during a ZIKV outbreak on Yap Island, rash was the most reported complaint (90% of cases), alongside arthritis and arthralgia (65% of cases) [1].

Our study has some important limitations. As a cross-sectional study, it is difficult to establish causality, and further prospective studies will be required in the future. Additionally, we collected data about the pregnancy retrospectively, which creates a risk of memory bias. We took careful steps to reduce this memory bias, and interviews were performed by trained health professionals who used the subject’s medical record and prenatal Brazilian monitoring program card to reduce bias. Another study limitation was that we did not collect blood samples for all participants at the same time. That said, we found a high concordance (93%) of PRNT results between the samples from the delivery period and those performed at follow up (2 to 3 years later). This finding is important in order to assess the exposure to ZIKV after the outbreak, a finding which may be a valuable tool for epidemiological surveillance actions and which may help to provide guidance for women in childbearing age about ongoing risk of exposure to the virus. Finally, PRNT results for the four DENV serotypes were available only for 23% of the recruited mothers. Still, when we compared DENV infection rates, between our ZIKV positive and negative subjects, we did not find any statistically significant differences (S2 Table), suggesting that environmental exposure to disease-carrying arthropod vectors may not have varied substantially between groups.

Social determinants of health are factors associated with an increased risk of contracting a wide range of diseases [39]. ZIKV is a worldwide public health issue, however, as in the case of dengue and other mosquito-borne infections, the risk of ZIKV infection can be linked to several primary sociodemographic determinants. We identified three key factors: low education, food insecurity, and younger age which are associated with the risk of ZIKV infection in a vulnerable population located in an undeveloped region. We believe that these findings may facilitate prediction of areas and individuals who may be at a particularly elevated risk of infection. Furthermore, we hope that these findings can be combined with pre-existing knowledge about environmental factors such as mosquito prevalence, which are also major risk factors for infection with arthropod-borne diseases like ZIKV and DENV to create public health campaigns that focus on reducing social inequalities in addition to enacting vector control and other basic environmental interventions.

## Supporting information

S1 Data Excel spreadsheet containing, in separate spreadsheets, the data used in tables and figures. (XLS)

S1 Table Food insecurity characteristics among pregnant women whom were categorized at risk in the screening. (DOCX)

S2 Table Comparison of available DENV PRNT results between the pregnant women with a positive ZIKV PRNT and pregnant women with negative ZIKV PRNT results. (DOCX)
