# Supplementary material for: Social determinants associated with Zika virus infection in pregnant women
Source: PLoS Negl Trop Dis. 2021 Jul 30;15(7):e0009612. doi: 10.1371/journal.pntd.0009612 (PMC8323902; doi:10.1371/journal.pntd.0009612)
Supplement: S1 Table — (DOCX) [file pntd.0009612.s002.docx]

**S1 Table. Food insecurity characteristics among pregnant women whom were categorized at risk in the screening**

| **Characteristic** | **No. responses** | **Total**  **(N=247)^a^** | **Zika positive, (N=158)^a^** | **Zika negative, (N=89)^a^** | **p-value** |
| --- | --- | --- | --- | --- | --- |
| Unable to offer children healthy diet | 246 | 129 (52) | 83 (53) | 46 (52) | 0.89 |
| Reduction or food restriction due to lack of money | 246 | 167 (68) | 106 (68) | 61 (69) | 0.89 |
| Weight loss due to not having money for food | 247 | 40 (16) | 28 (18) | 12 (13) | 0.47 |
| Not enough resources to have a healthy and varied diet | 247 | 157 (64) | 100 (63) | 57 (64) | >0.99 |
| Children did not eat enough due to lack of money | 246 | 63 (26) | 39 (25) | 24 (27) | 0.76 |
| Reduced meal portion sizes or skipped meals due to lack of money | 245 | 118 (48) | 74 (47) | 44 (50) | 0.69 |
| Eat less than you should because you do not have enough money | 247 | 120 (49) | 81 (51) | 39 (44) | 0.29 |
| Went without eating for a whole day or just had one meal because you do not have money | 243 | 48 (20) | 31 (20) | 17 (20) | >0.99 |
| Reduced the portion size of your children's meals due to lack of money | 247 | 41 (17) | 27 (17) | 14 (16) | 0.86 |
| Children have already skipped a meal because they don't have enough money | 245 | 21 (8.6) | 12 (7.6) | 9 (10) | 0.48 |
| ^a^n (%) | | | | | |
