# Supplementary material for: Social determinants associated with Zika virus infection in pregnant women
Source: PLoS Negl Trop Dis. 2021 Jul 30;15(7):e0009612. doi: 10.1371/journal.pntd.0009612 (PMC8323902; doi:10.1371/journal.pntd.0009612)
Supplement: S2 Table — (DOCX) [file pntd.0009612.s003.docx]

**S2 Table. Comparison of available DENV PRNT results between the pregnant women with a positive ZIKV PRNT and pregnant women with negative ZIKV PRNT results.**

|  | **Responses (N=469)^a^** | **Zika positive (N=287)^a^** | **Zika negative (N=182)^a^** | **p-value** |
| --- | --- | --- | --- | --- |
| **DENV 1 PRNT_50_** | 106 |  |  | 0.082 |
| Positive | 76 (72) | 61 (76) | 15 (58) |  |
| Negative | 30 (28) | 19 (24) | 11 (42) |  |
| **DENV 2 PRNT_50_** | 109 |  |  | 0.51 |
| Positive | 61 (56) | 47 (58) | 14 (50) |  |
| Negative | 48 (44) | 34 (42) | 14 (50) |  |
| **DENV 3 PRNT_50_** | 104 |  |  | >0.99 |
| Positive | 63 (61) | 47 (60) | 16 (62) |  |
| Negative | 41 (39) | 31 (40) | 10 (38) |  |
| **DENV 4 PRNT_50_** | 107 |  |  | 0.38 |
| Positive | 60 (56) | 47 (59) | 13 (48) |  |
| Negative | 47 (44) | 33 (41) | 14 (52) |  |
| **DENV 1 PRNT_90_** | 106 |  |  | 0.068 |
| Positive | 59 (56) | 49 (61) | 10 (38) |  |
| Negative | 47 (44) | 31 (39) | 16 (62) |  |
| **DENV 2 PRNT_90_** | 109 |  |  | 0.19 |
| Positive | 47 (43) | 38 (47) | 9 (32) |  |
| Negative | 62 (57) | 43 (53) | 19 (68) |  |
| **DENV 3 PRNT_90_** | 104 |  |  | 0.80 |
| Positive | 27 (26) | 21 (27) | 6 (23) |  |
| Negative | 77 (74) | 57 (73) | 20 (77) |  |
| **DENV 4 PRNT_90_** | 107 |  |  | 0.27 |
| Positive | 22 (21) | 19 (24) | 3 (11) |  |
| Negative | 85 (79) | 61 (76) | 24 (89) |  |
| ^a^n (%)  DENV - Dengue Virus, PRNT - Plaque Reduction Neutralization Test | | | | |
